# The effect of azoospermia factor microdeletions on intracytoplasmic sperm injection results in azoospermia patients

**DOI:** 10.12669/pjms.39.3.7003

**Published:** 2023

**Authors:** Volkan Emirdar, Ferruh Acet

**Affiliations:** 1 Volkan Emirdar, MD, Department of Obstetrics and Gynecology, Izmir Economy University, Medicalpoint Hospital, Izmir, Turkey; 2 Ferruh Acet, MD, Department of IVF Research and Training Center, Ege University Faculty of Medicine, Izmir, Turkey

**Keywords:** Azoospermia, ICSI, Pregnancy, Infertility, Microdeletion

## Abstract

**Background &Objective:**

Y chromosome abnormalities are common in male patients with severe oligo-azoospermia. In studies with karyotype analysis and cytogenetic methods, the importance of the Y chromosome in spermatogenesis has been well understood. Deletions in the azoospermia factor (AZF) localized at the distal end of the Y chromosome adversely affect the spermatogenesis process. Our objective was to determine the frequency of AZF microdeletion in azoospermia patients who underwent microTESE.

**Methods:**

In this retrospective cohort study, 806 azoospermic men attending the In Vitro Fertilization (IVF) Center for infertility treatment between 2010 and 2022 were included. AZF deletion screening was conducted in all patients included in the study. Azoospermic patients with and without Y microdeletion were matched with the female’s age, cause of infertility, number of oocytes retrieved and number of metaphase II (MII) oocytes produced and compared. The primary outcome was the live birth rate (LBR). Pregnancy rate (PR) and clinical pregnancy rates (CPR) were secondary outcomes.

**Results:**

We detected Y microdeletion in 55 (6.82%) of 806 infertile azoospermic men and 35 of them included in the study. Although the required gonadotropin dose and the total number of retrieved oocytes were similar, clinical pregnancy rates and live birth rates were found to be significantly lower in the microdeletion patient group (21.6% vs. 43%, p<0.05; and 18.9% vs. 36%, p<0.05, respectively).

**Conclusions:**

Poor sperm quality in AZF microdeletion patients complicates the selection of appropriate sperm for ICSI. Therefore, it leads to a decrease in embryonic development, fertilization and pregnancy results. In order to select the best sperm for the use in ICSI procedure in this patient population, intracytoplasmic morphologically selected sperm injection (IMSI) method can be preferred to improve the cycle outcomes.

## INTRODUCTION

The incidence of infertility among couples varies between 9% to 15% and it is known that this situation affects the quality of life, anxiety and stress levels of infertile couples. 1,2 Among etiologic factors; female factor is responsible for 40-50%, male factor 30%, while male and female factors together exist 20% of the etiologic factors.^3^ Varicocele, infections, genetic disorders, congenital abnormalities, drug use, radiotherapy, chemotherapy, metabolic and systemic diseases, ejaculatory duct obstructions and lifestyle habits can be listed among non-idiopathic male factor-related causes.

Even about 12% of the cases with non-obstructive azoospermia have abnormal karyotype, genetic testing is strongly recommended during etiology work up.^4^ In patients with severe oligoasthenoteratospermia, frequency of Y microdeletions is around 6-18%.^5^ Tiepolo, in 1976, in his karyotype analysis studies, determined that the Y chromosome has an important role in the spermatogenesis process.^6^ The same researchers determined that there was a deletion in the distal part of the Y chromosome in the karyotype analysis of patients to whom semen analysis had no sperm. This detected chromosomal location is called “Azoospermia Factor” region, carries the genes which play a role in sperm production process. Although having a normal phenotypic appearance, 10-20% of men with idiopathic infertility lack some of the azoospermia factor (AZF) regions on the long arm of the Y chromosome, which is necessary for fertility. These regions, which cannot be identified cytogenetically, are called AZFa, AZFb, and AZFc. The AZF region is located in the 11.23 region of the long arm of the Y chromosome. Cytogenetic and molecular studies have revealed that Y chromosome deletions are associated with abnormal spermatogenesis.^7^

In our patient group, sperm retrieval with testicular sperm extraction (TESE) and subsequent intracytoplasmic sperm injection (ICSI) can yield successful fertilization and pregnancy results.^8^ The aim of this single tertiary center study was to determine the frequency of AZF microdeletion in azoospermia patients who underwent microTESE. In addition, the results of assisted reproductive technology (ART) in this patient group were also evaluated.

## METHODS

For the study, 806 azoospermic men attending the In Vitro Fertilization (IVF) Center for infertility treatment between 2010 and 2022 were included. All patients were screened for Y chromosome microdeletions in the Department of Genetics. Semen analyzes were performed according to the World Health Organization criteria (WHO 2010).^9^ All patients included in the study filled out a questionnaire about whether they used cigarettes, alcohol, drugs or any other substance. The history and coitus characteristics of the patients were questioned in detail. A general testicular examination was performed. Chromosomal karyotype analysis was performed using G-band staining of peripheral blood lymphocytes and all subjects had normal karyotype.

Azoospermic patients without Y microdeletion were selected as the control group. The control group of patients were matched with the female’s age, cause of infertility, number of oocytes retrieved and number of metaphase II (MII) oocytes produced. Consent form was obtained from all patients in the study. Ethics Committee approval was obtained for the study (25.04.2022-E.664708).

The inclusion criteria were patients under the age of 40 who underwent ICSI due to azoospermia. Cases with congenital uterine abnormality, myoma uteri, hydrosalpinx, Asherman Syndrome, advanced maternal age (>40), endometrial polyp and thrombophilia were excluded from the study.

Live birth rate was the primary outcome of the study. Clinical pregnancy rate (intrauterine sac seen on transvaginal ultrasonography) and pregnancy rate (human chorionic gonadotropin beta subunit (B-hcg) >50 u/L on 14 days after embryo transfer) were secondary outcomes of the study.

### Semen analysis:

Semen samples from all men included in the study were evaluated according to the WHO criteria.^8^ The ejaculates were taken after three to five days of sexual abstinence, and counted using a Makler Chamber. In azoospermic men no sperm was observed in the pellet, under the inverted microscope after centrifugation at 2500g. Azoospermia was diagnosed when no sperm were detected in at least two spermiogram performed with an interval of 15 days in all patients whose hormonal measurements were made. “Testicular Sperm Extraction” (TESE) was performed to the patients who were found to have azoospermia in the semen analysis to investigate the presence of sperm for the treatment of infertility. Tubules taken from the testicular tissue by incision were dissected under a stereomicroscope and examined for the presence of sperm under an inverted microscope.

### Methods for Detection of Y Chromosome Microdeletion:

Diagnosis of microdeletions in the Y chromosome was performed by capillary electrophoresis with ChromoQuant AZF PCR (Solna) kit. Genomic DNA was extracted from peripheral blood samples using DNA extraction kit (QIAamp DNA Blood Mini Kit, QIAGEN, Valencia, CA), and amplified in multiplex polymerase chain reaction (PCR). After the PCR step, capillary electrophoresis was performed on the amplified DNA regions on the ABI Prism 3,130 genetic analyzer. Each of these subjects was tested for seven AZF loci: the primers used for AZFa (sY84, sY86), AZFb (sY127, sY134) and AZFc (sY254, sY255, sY160 heterochromatin). The internal controls were SRY (Yp11.3) and ZFX/Y (Yp11.31 Xp 21.3). The PCR setup was carried out according to the content of the kit. Amplification was carried out in the following thermal profile: initial denaturation at 95ºC for 20 min followed by 30 cycles of denaturation at 94ºC for 60 s, annealing at 63ºC for 90 s, extension at 73ºC for 60 s, followed by a final extension at 70ºC for 20 min. The interpretation was made according to the EAA / EMQN guideline.^10^

### In Vitro Fertilisation Protocol:

Antagonist protocol was preferred for ovarian stimulation for all patients included in the study. On the third day of the cycle, the ovarian reserve was evaluated and 150-375 IU gonadotropin (follitropinalfa and/or menotropin) treatment was started, and on the 7th day, Cetrorelix (Cetrotide, Merck, Halle, Germany) treatment was started. Human chorionic gonadotropin (hCG) was administered for final oocyte maturation triggering, oocyte retrieval was performed 36 hours after the trigger. Oocyte cumulus complexes were then incubated for two hours in SAGE one-step with a human serum albumin (HSA) medium (Origio, Denmark) at 6% CO_2_, 5% O_2_ and 37°C before oocyte denudation. Mechanical pipetting in 10-IU/mL hyaluronidase (HYASE-10×; Vitrolife, Sweden) was applied for the detachment of the cumulus cells. The description of the first polar body was used for the evaluation of the nuclear maturation. Intracytoplasmic sperm injection (ICSI) was performed in Quinn’s advantage medium containing HEPES (plus HSA; Cooper). Oocytes after ICSI were cultured individually in 20 µl dome-shaped microdrop of Sage one-Step (Origio, Denmark) culture media in petri dish (Nunc IVF Petri Dish 150270, Thermo Scientific) covered by liquid paraffin in the standard incubator in an atmosphere of 5.5% CO_2_ and %5.0 O_2_. Fertilisation was assesed at 16-18 hour after intracytoplasmic sperm injection by the presence of two nuclei. Cleaving embryos were scored and graded according to the numbers of blastomeres, their symmetry and degree of cytoplasmic fragmentation as described by Veeck et al.^11^ On day five or day six blastocysts were classified according to Gardner’s score.^12^ Data were collected from patients’ medical charts.

### Statistical analysis:

All statistical analyses were carried out with the Statistical Package for the Social Sciences version 15.0 (SPSS Inc, Chicago, IL, USA). Differences between the means in normally distributed variables were compared by using Student’s t-test. Chi-square test was carried out on categorical variables. A p value less than 0.05 was considered statistically significant.

## RESULTS

**Fig.1:**
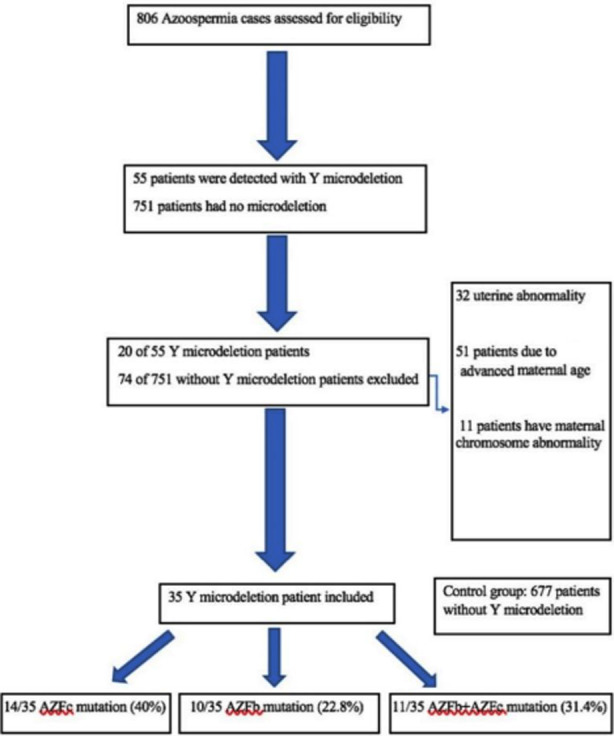
Flowchart of the study.

We detected Y microdeletion in 55 (6.82%) of 806 infertile azoospermic men in our patients. Out of 806 patients, 94 were excluded (32 patients had uterine abnormality, 51 patients due to advanced maternal age, 11 patients had maternal chromosome abnormality) from the study according to the exclusion criteria. In total, 35 azoospermic patients with Y microdeletion and 677 patients for the control group were included in this retrospective study (Fig.1).

Mutation in the AZFc region was detected in 14 (40%) of the cases, while AZFb and AZFc mutations were detected in 11 (31.4%) cases. Only AZFb microdeletion was observed in 10 (%22.8) of the cases with microdeletion. (concomitant chromosomal abnormality) (Table-I).

**Table-I:** Distribution of AZF microdeletion in patients.

AZFc	14 ( 40%)
AZFb	10 (22,8 %)
AZFb + AZFc	11 (31.4%)

Values are n (%).

Basic characteristics such as age, body mass index, duration of infertility and anti-mullerian hormone (AMH) levels were similar in both groups (Table II). The comparison of IVF cycle characteristics and pregnancy outcomes in both groups is shown in Table-III. Although the required gonadotropin dose and the total number of oocytes retrieved were similar, clinical pregnancy and live birth rates were found significantly lower in the microdeletion patient group (21.6% vs. 43%, p<0.05 and 18.9% vs. 36%, p<0.05, respectively).

**Table-II:** Baseline characteristics of the groups.

	AZF microdeletion (n:35)	Control (n:677)	p
Age (years)	31.4 ± 3.9	32.8 ± 3.7	0.65
BMI (kg/m2)	24.6 ± 3.1	24.5 ± 3.3	0.80
Duration of infertility (years)	2.2 ± 1.1	2.4 ± 1.6	0.75
AMH	2.1 ± 1.9	2.2 ± 1.8	0.63

Values are mean ± SD (95% CI). BMI, body mass index; AZF, azoospermia factor; AMH, Antimullerian hormone.

**Table-III:** Cycle characteristics and IVF outcomes of the groups.

	AZF microdeletion (n:35)	Control (n:677)	p-value
Total dose of gonadotropins (IU)	2276.6 ± 803.5	2360.0 ± 816.5	0.62
Number of retrieved oocytes	11.5 ± 8.77	11.92 ± 5.79	0.75
Number of MII oocytes	9.09 ± 7.57	12.23 ± 6.56	0.43
Fertilisation rate (%)	51	74	<0.005
Embryo cleavage rate^a^ (%)	75	91	0.035
Grade 1-2 embryo rate (%)	41.58	51.2	0.025
Clinical pregnancy rate (%)	21.6	43	<0.005
Live birth rate (%)	18.9	36	0.015

a Percentage of the total number of embryos, Variables (Mean ± SD). AZF, azoospermia factor; IVF, in vitro fertilization.

## DISCUSSION

In our study, microdeletions on the Y chromosome of the AZF region, which controls spermatogenesis, was investigated in azoospermic men. We detected Y microdeletion in 6.82% of 806 infertile azoospermic men, which is consistent with the findings of the previous studies.^13^,^14^ Although live birth rates of 36% were achieved in the control group, the results were almost half as bad in patients with Y microdeletion. When the literature is reviewed, studies show that ICSI results of patients with microdeletion and patients with normal karyotype are similar in azoospermic men.^15^,^16^ However, in our study although the groups had similar basic characteristics and similar oocyte numbers; fertilization rates, clinical pregnancy and live birth rates were significantly lower in the Y microdeletion group.

With the development of assisted reproductive techniques and especially the introduction of intracytoplasmic sperm injection technique, sperm can be obtained and directly injected into the cytoplasm of the oocyte to yield better outcome especially in severe oligoasthenozoospermia and azosparmia patients.^17^ Thus, high pregnancy results can be achieved in azoospermic men thanks to the micro TESE method.^18^

AZF mutations are the most common region of Y microdeletions in cases of non-obstructive azoospermia or severe oligoasthenoteratospermia. The highest rate of these microdeletions is in the AZFc region. Less frequently (1-5%), microdeletions are detected in the AZFb region.^10^ In our study, similar to most of the other publications in the literature, AZFc mutation was observed in 40% of the cases while isolated AZFb mutations were observed in 22.8% and combined mutations were observed in 31.4% among our patients. The incidence of AZFbc is higher in our country than in European countries and similar to Asian countries.^19^,^20^ Although there are publications about the low rate of sperm retrieval with TESE in men with AZFb mutations, eligible sperm for ICSI were found in two cases in our study group.^21^, ^22^ There is very limited data on IVF outcomes in cases with AZFb mutations. Fertilization and embryo cleavage development was observed in one of two cases, resulting in a live birth. Men with AZFb deletions usually fail to yield spermatozoa by using testicular sperm extraction. In the literature, it has been shown that sperm can be obtained in these cases, especially when there is only partial AZFb mutation detected.

Controversial results are seen in the literature regarding the effect of AZF mutation on IVF/ICSI outcomes. Although there are articles stating that it does not affect cycle outcomes, there are also studies reporting impaired pregnancy outcomes in this patient group.^23^,^24^ Our results are consistent with those of these previous researchers who found decreased pregnancy rates in Y microdeletion patients. In our study, the female infertility evaluations were completely normal. The only factor in the etiology of infertility was male azoospermia. Therefore, we thought that a homogeneous evaluation was made, and we found that pregnancy outcomes were statistically significantly lower in the presence of Y microdeletion (live birth rate 18.9% v. s 36%; p<0.05). The strength of our study is that the live birth rates are given for the two groups, which is the most important outcome for predicting IVF success when the female age and ovarian reserve are similar.

One of the major concerns in AZF microdeletion patients is the defective sperm production leading to incapable sperm production with advancing age. AZFc gene location is essential for the function of genes that play an important role in the spermatogenesis process; therefore, AZFc microdeletions cause failures in the spermatogenesis process. Partial deletions of the genes chromo domain Y (CDY) and deleted in azoospermia (DAZ) in the AZFc region lead to reduced sperm count and motility and then showed severe oligozoospermia or even azoospermia.^24^ Sperm maturation defect and meiosis aberrations occurring in the seminiferous ducts cause a small amount of mature sperm. There are even clinics that offer sperm cryopreservation at young age to these patients for the risk of future severe male factor infertility. However, in this study, sperm were retrieved in all cases with micro TESE and these patients were given the chance to become fathers of their biological offspring.

The possibility of father-to-son transmission of the Y microdeletion is another interesting topic. These patients in our clinic were consulted with the genetics department and about the possibility of this transmission. However, preimplantation genetic screening is not performed, as a somatic effect of this deletion has not been showed in children before.^25^ Yet, it should be explained to all patients that if this mutation is transmitted to a male baby, they may experience infertility problems in their future life.

Although it was previously shown that the basal hormone profiles (follicle-stimulating hormone, luteinizing hormone, androgens) and testicular volumes did not affect the ICSI results, the parameters of the patients included in the study were similar.^17^

When the literature is examined, it has been shown that the embryo quality obtained after ICSI is not affected when sperm are used from a Y chromosome microdeletion positive patient.^26^ As a result of our study with a high number of participants, different from the literature, fertilization rates and good quality embryo rates were statistically significantly lower in men with Y microdeletion. Significantly lower rates of clinical pregnancy and live birth rates were found in azoospermia cases with microdeletion when compared to azoospermia cases with normal karyotype. Sharing the fact that the results can be lower in this patient group while giving counseling in the light of the literature will allow better understanding for the patients.

### Limitations:

Not to have miscarriage rates of the groups and retrospective design of our study are the limitations of our study. On the other hand, strength of our study is having the data of live birth rates for each group in quite large sample size study.

Currently, ICSI brings new hopes for azoospermic patients who want to have children with their own gametes and can be applied to men with a Y chromosome microdeletion in IVF treatments. However, while counseling this patient group before treatment, it should be noted that Y microdeletion may adversely affect the treatment results.

## CONCLUSION

In conclusion; poor sperm quality in AZF microdeletion patients complicates the selection of appropriate sperm for ICSI. In this case, it may lead to a decrease in embryonic development, fertilization and pregnancy outcomes. In order to select the best sperm for the use in ICSI procedure in this patient population, intracytoplasmic morphologically selected sperm injection (IMSI) method can be preferred. This improved selection method can optimize the ICSI outcome for AZF patients.
